# Field-Effect Transistor Biosensor for Rapid Detection of Ebola Antigen

**DOI:** 10.1038/s41598-017-11387-7

**Published:** 2017-09-08

**Authors:** Yantao Chen, Ren Ren, Haihui Pu, Xiaoru Guo, Jingbo Chang, Guihua Zhou, Shun Mao, Michael Kron, Junhong Chen

**Affiliations:** 1 Department of Mechanical Engineering, University of Wisconsin-Milwaukee, 3200 N. Cramer Street, Milwaukee, WI 53211 USA; 2 Tianjin Key Laboratory for Photoelectric Materials & Devices, School of Materials Science and Engineering, Tianjin University of Technology, Tianjin, 300384 P.R. China; 3 State Key Laboratory of Pollution Control and Resource Reuse, School of Environmental Science and Engineering, Tongji University, 1239 Siping Road, Shanghai, 200092 P.R. China; 4 Department of Medicine, Division of Infectious Diseases, Biotechnology and Bioengineering Center, Medical College of Wisconsin, 8701 Watertown Plank Road, Milwaukee, WI 53226 USA

## Abstract

The Ebola virus transmits a highly contagious, frequently fatal human disease for which there is no specific antiviral treatment. Therefore, rapid, accurate, and early diagnosis of Ebola virus disease (EVD) is critical to public health containment efforts, particularly in developing countries where resources are few and EVD is endemic. We have developed a reduced graphene oxide-based field-effect transistor method for real-time detection of the Ebola virus antigen. This method uses the attractive semiconductor characteristics of graphene-based material, and instantaneously yields highly sensitive and specific detection of Ebola glycoprotein. The feasibility of this method for clinical application in point-of-care technology is evaluated using Ebola glycoprotein suspended in diluted PBS buffer, human serum, and plasma. These results demonstrate the successful fabrication of a promising field-effect transistor biosensor for EVD diagnosis.

## Introduction

The 2014–2015 outbreak of Ebola virus disease (EVD) in West Africa resulted in thousands of deaths and generated worldwide panic. The Ebola virus is highly contagious and is transmitted between humans through contact with infected body fluids^1–4^. The initial symptoms of Ebola virus infection are non-specific, yet early diagnosis is critically important to public efforts to contain this disease, which has reported fatality rates of 30–90%^5^. In July 2016, the U.S. Food and Drug Administration granted breakthrough therapy designation to an investigational Ebola vaccine manufactured by Merck, known as V920^6^; however, as yet there are no specific antiviral medicines proven effective against the Ebola virus.

The recent EVD epidemic also has shown that even diagnostic methods based on reverse transcription polymerase chain reaction (RT-PCR) technology^7^ can yield false negative results within the first few days of an infection due to the very low levels of viremia present at the onset of symptoms. The longer diagnosis is delayed, the greater the amount of virus present in patients’ body fluids and thus the risk of contagion rises accordingly. EVD also can demonstrate an incubation period of up to 21 days, and thus diagnostic assays often must be performed. Several diagnostic methods have been studied for rapid diagnosis of EVD, such as lateral flow chromatography^8, 9^, single-particle interferometric reflectance imaging sensors^10^, opto-fluidic chips^11^, and opto-fluidic nanoplasmonic biosensors^12^. In 2015 the World Health Organization approved the ReEBOV Antigen Rapid Test Kit (Corgenix, USA) for use under emergency authorization^13^, yet there remains an urgent, unmet demand for rapid, sensitive, specific, and low-cost diagnosis of EVD.

Field-effect transistor (FET) is an attractive platform for the rapid and accurate detection of various analytes. For example, its utility has been demonstrated for detecting target analytes in gases^14–17^ and in water^18, 19^. The advantages of FET sensors include fast response, low-cost, and ease of use, because the real-time results are monitored with low-cost meters that can be calibrated for different applications. FET biosensors can achieve high sensitivity and selectivity for specific biomolecules by anchoring specific probes on the conducting channel^20–23^, which is a critical factor for FET sensor performance. Two-dimensional (2D) semiconductor materials, such as graphene, MoS_2_, and black phosphorous (BP), are particularly attractive as conducting channels for FET biosensors because of their superior electronic properties. Mao *et al*. reported that reduced graphene oxide (rGO) was useful as a conducting channel in FET biosensors for detecting human immunoglobulin G^20, 22^. Xu *et al*. reported that graphene fabricated by chemical vapor deposition (CVD) can be used in the FET biosensor for detection of adenosine triphosphate^24^ and determination of binding kinetics of DNA hybridization^25^. Huang *et al*. also reported that graphene films obtained by CVD can be used in detection of bacteria^26^. In order to be used to diagnose EVD, the FET biosensor should have a sensitivity close to that of PCR, a lower per-test cost, and minimum investment in the necessary laboratory hardware.

The Ebola virus surface glycoprotein (GP) GP_1,2_ is an important virulence factor for the production of Ebola hemorrhagic fever and is present in all five species of Ebola virus: Zaire, Sudan, Cote d’Ivore, Reston, and Bundibugyo^27^. GP_1,2_ plays a role in disease pathogenesis by mediating endothelial cell dysfunction and immune suppression^28–30^. Attachment of the Ebola virus to host cell surfaces is mediated by the lectin binding of GP_1,2_, fusion with host cells, and, eventually, secretion of large quantities of a truncated glycoprotein, sGP. The identification of a large number of viral particles in humans infected with the Ebola virus suggests that both antigen detection and viral RNA detection systems could be effective for diagnosis, depending on the sensitivity of the assay^31^. Research by Ksiazek *et al*. demonstrated that Ebola virus antigenemia and virus infectivity assays (i.e., viral titers) correlated well, particularly when a minimum virus threshold of 10^2^ to 10^3^ pfu/ml was exceeded^32, 33^.

In this article, we report the development of an FET biosensor in which rGO instead of MoS_2_ or BP is used as the conducting channel, considering the cost and stability of rGO for the practical application. rGO-based FET devices can be easily obtained by thermal annealing of graphene oxide (GO) sheets that are chemically adsorbed on the electrode, with extra advantages of low-cost and experimental flexibility, compared with graphene synthesized by the CVD method. The channel has immobilized anti-Ebola probes that selectively capture the antigen. We found that such an FET biosensor has high selectivity and sensitivity towards the Ebola glycoprotein (EGP) of the Zaire strain, with a limit of detection down to 1 ng/ml. To the best of our knowledge, this is the first report describing Ebola antigen detection using an FET biosensor. Our work demonstrates the great potential of using an FET-based platform in the early diagnosis of pestilence.

## Results

**Figure 1:**
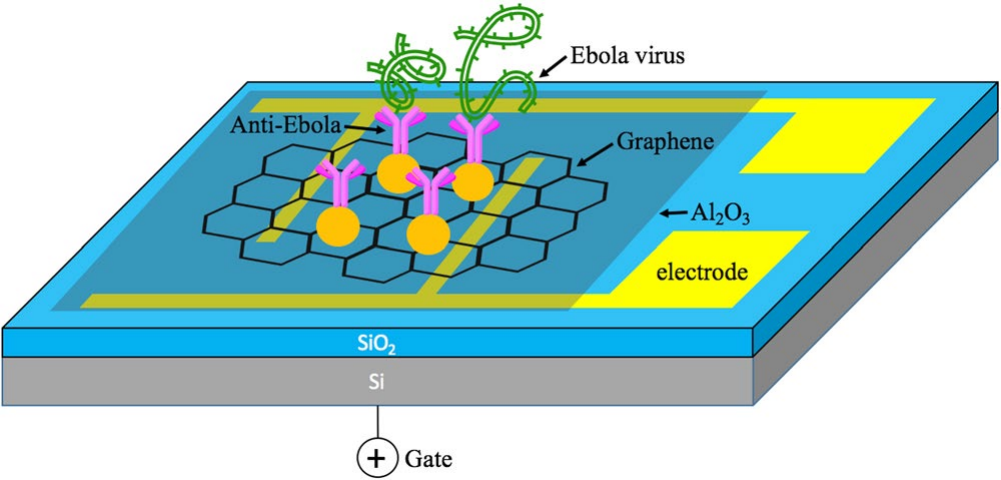
Schematic diagram of the rGO-based FET biosensor. rGO sheet was deposited onto the device to bridge the drain and the source electrode. Al_2_O_3_ was coated on the rGO sheet for surface passivation. Ebola antibodies were conjugated with gold NPs on the channel and functioned as sensing probes for capturing Ebola antigens.

**Figure 2:**
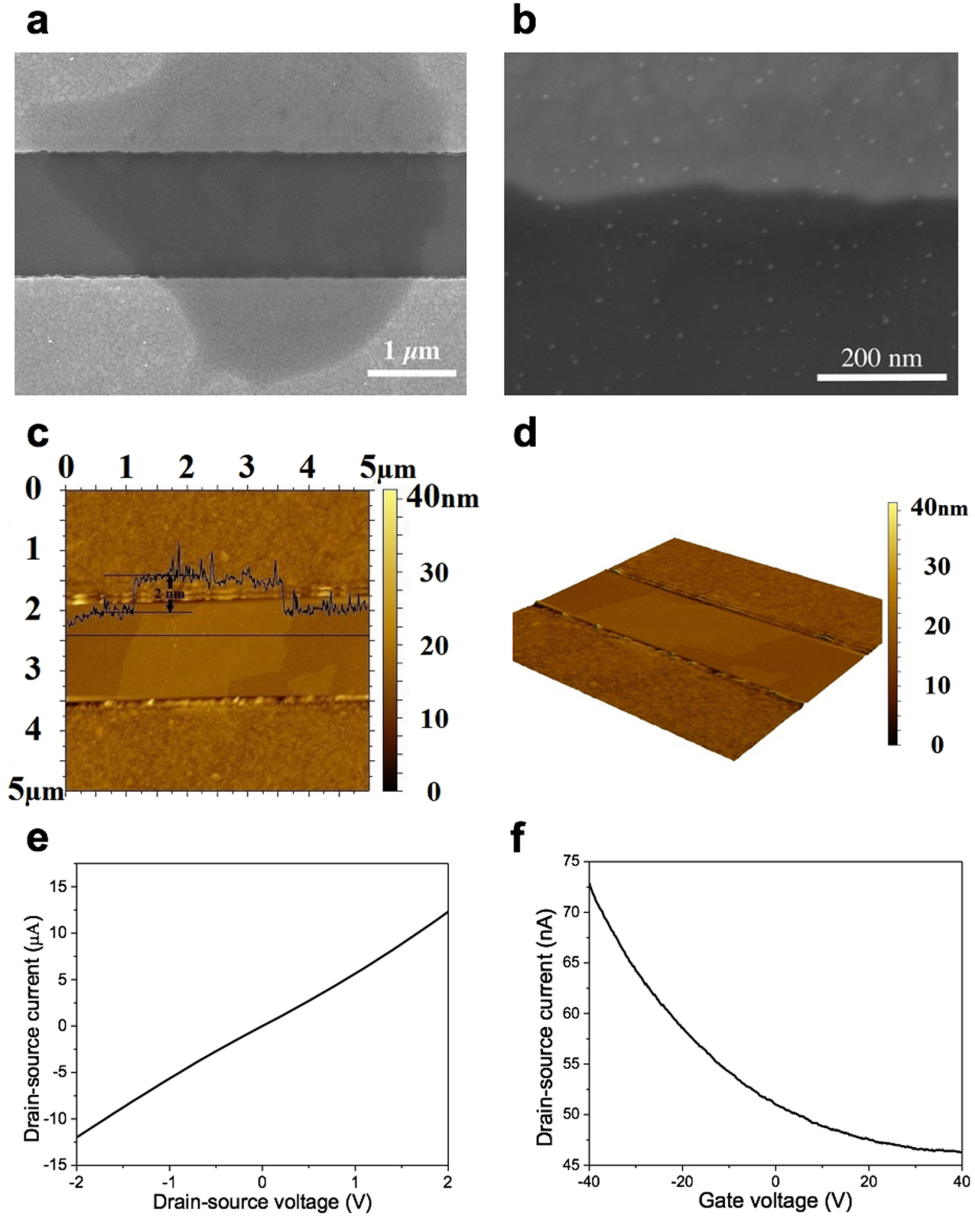
FET biosensor characterization. (**a**–**d**) SEM and AFM images of the rGO-based FET biosensor. (**a**) One piece of rGO sheet bridging the source and the drain electrodes. (**b**) SEM image of the rGO sheet with a higher magnification. Gold NPs were found to be uniformly distributed on the rGO sheet. (**c**) The thickness of rGO sheet was measured as ~ 2 nm by AFM. (**d**) 3D view of an rGO sheet across the electrodes. (**e**,**f**) Direct current and transistor measurements on one piece of the FET device. (**e**) I-V curve of the FET device. A linear characteristic without significant bent was observed, indicating the Ohmic contact between the rGO sheets and electrodes. (**f**) Transistor measurement was performed with a fixed V_ds_ (0.01 V). rGO sheets show a typical p-type semiconductor nature (on-off current ratio ~ 1.5) under the ambient condition.

Figure 1 shows a schematic diagram of the rGO-based FET device with the Ebola antibody immobilized to detect the specific antigen. Gold nanoparticles (NPs) were electrosprayed on the rGO sheet and chemically functionalized for antibody conjugation. Scanning electron microscopy (SEM) was used to investigate the structure of the as-produced FET device. In Fig. 2a, one piece of rGO sheet with the surface passivated by Al_2_O_3_ film was found to bridge the gap between the drain and source electrodes. An SEM image with a higher magnification is shown in Fig. 2b. The gold NPs were deposited uniformly on the device by sputter coating for antibody conjugation. The gold NPs could not be observed before sputter coating, indicating these bright “dots” are gold NPs produced by sputter coater. To further characterize the FET device, Atomic force microscopy (AFM) was conducted to measure the thickness of the rGO sheet. As shown in Fig. 2c, the rGO sheet has a thickness around 2 nm, indicating the few-layer structure, while the monolayer thickness is 0.34 nm. A 3D view of an rGO sheet is shown in Fig. 2d to better understand the device structure.

**Figure 3:**
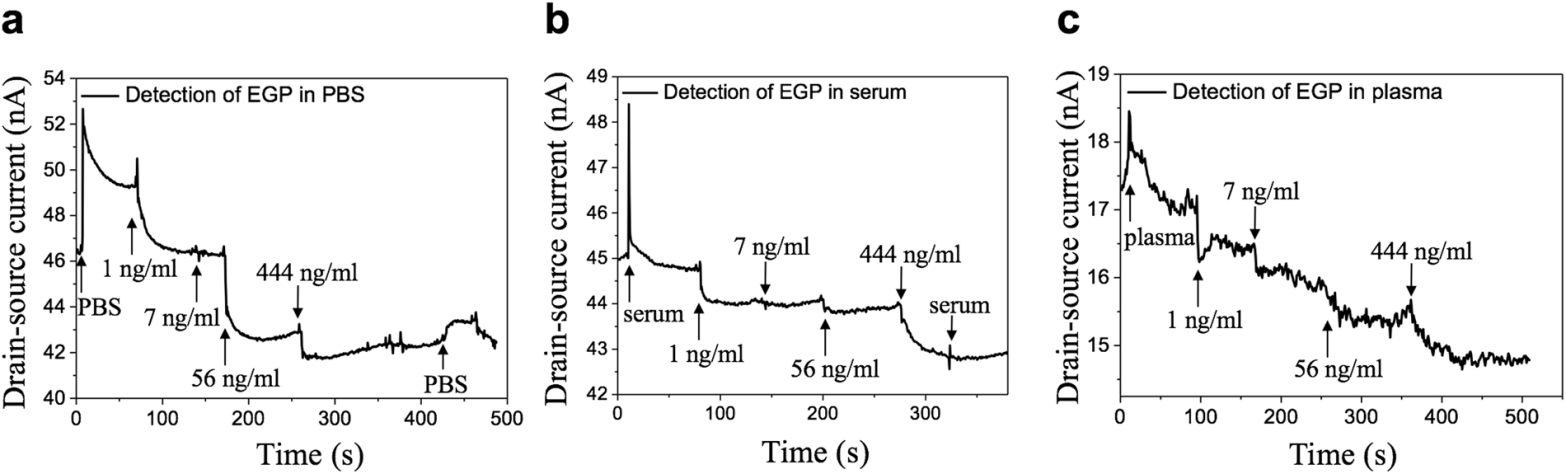
Dynamic response of the FET biosensor towards EGP. (**a**) EGP suspended in 0.01 × PBS. (**b**) EGP suspended in 0.01 × human serum. (**c**) EGP suspended in 0.01 × human plasma. The sensor’s response to EGP is instantaneous. For (**a**–**c**), V_ds_ was fixed at 0.01 V.

Direct current measurement was conducted to study the electrical characteristics of the FET biosensor. The I-V curve is shown in Fig. 2e. The typical resistance of the FET devices ranges from 10^4^ Ω to 10^6^ Ω. The I-V curve shows a linear characteristic, which demonstrates the Ohmic contact between the rGO sheets and the electrodes. The transistor measurement was conducted to characterize the semiconductor nature and the on-off current ratio. A fixed drain-source voltage (V_ds_) of 0.01 V was applied between the drain and source electrodes with gate voltage (V_g_) sweeping from −40.0 V to + 40.0 V. The obtained FET transfer curve in Fig. 2f indicates that the rGO sheet has a typical p-type semiconductor nature with an on-off ratio ~1.5. To investigate the sensor performance, the sensor dynamic response towards EGP was studied with a fixed V_ds_ (0.01 V) across the drain and source electrodes, while V_g_ was set at zero. A higher V_ds_ may result in a noisier response and even the damage of rGO sheets. EGP was purchased from a commercial vendor (IBT Bioservices) in the form of purified protein and suspended in 0.01 × phosphate-buffered saline (PBS)/human serum/human plasma. The Debye length increases as the PBS/serum/plasma is diluted, so the surface charge carried by EGP is not screened, leading to an improved response^34^. For 0.01 × PBS, the corresponding Debye length is about 7.4 nm^35^, while the size of gold NPs is below 5 nm and the size of Ebola antibody and EGP is estimated by the vendor as 3-10 nm. As a result, the total size of gold NP plus antibody is likely within the Debye length of 0.01 × PBS, indicating the charges carried by antigen will not be completely screened. For the initial test, 0.01 × PBS was pipetted onto the device to create the baseline. After a stabilized signal was observed, samples with increasing EGP concentrations (1-444 ng/ml in 0.01 × PBS, 1 µL volume) were pipetted onto the sensor. Figure 3a shows the dynamic response of the FET biosensor towards EGP in 0.01 × PBS. The drain current (I_d_) dropped correspondingly as EGP concentration increased. We define the sensor’s sensitivity as ΔI/I_0_, where ΔI is the change in the I_d_ and I_0_ is the baseline current. According to the experimental result in Fig. 3a, as the concentration of EGP was elevated from 1 ng/ml to 444 ng/ml, the sensitivity increased correspondingly from 6.5% to 14%. It is noteworthy that the current change became less significant as the concentration of antigen increased. In other words, the sensitivity is not linearly increasing with the antigen concentration. This is because the total number of antibody probes on the channel is limited. As more probes are occupied by the antigen, a less significant response will be observed although the overall concentration of antigen keeps increasing. After testing the EGP samples, 0.01 × PBS was pipetted again onto the device. The current slightly increased and then recovered, indicating that the signal was generated by the protein binding rather than the buffer solution. Very importantly, the sensor had an instantaneous response to EGP within a few seconds.

Identifying the Ebola virus in early human infections is challenging with current technologies, especially for point-of-care applications, because the viral load initially may be undetectable, but rapidly multiply to fatal level. To investigate sensor performance under a more complicated but practical condition, we used 0.01 × human serum/plasma obtained from the Blood Center of Wisconsin to suspend EGP as a way to simulate blood samples from Ebola patients. As before, 0.01 × serum/plasma was drop-cast onto one piece of the device to establish the baseline, and then a group of EGP samples suspended in 0.01 × serum/plasma was pipetted onto the device. Figure 3b shows the sensor’s dynamic response to EGP in 0.01 × serum. The I_d_ dropped correspondingly with the increasing concentration of EGP. Then, 0.01 × serum was added again onto the biosensor and there was no significant change, confirming that only the specific binding can lead to a current change. The dynamic response towards EGP in 0.01 × plasma is shown in Fig. 3c with a much noisier signal. The response towards EGP in serum/plasma was similar to that in PBS but with a lower sensitivity (~1.7% for 1 ng/ml EGP in 0.01 × serum), indicating that the response is affected by the complex dispersion medium. The ionic strength of 0.01 × serum/plasma is 1.5 mM^34^, with corresponding Debye length close to that of 0.01 × PBS. The abundant non-specific proteins and other biomolecules^36^ contained in the serum/plasma sample would impact the recognition of the antigen, leading to a diminished response and sensitivity compared with that in the PBS.

**Figure 4:**
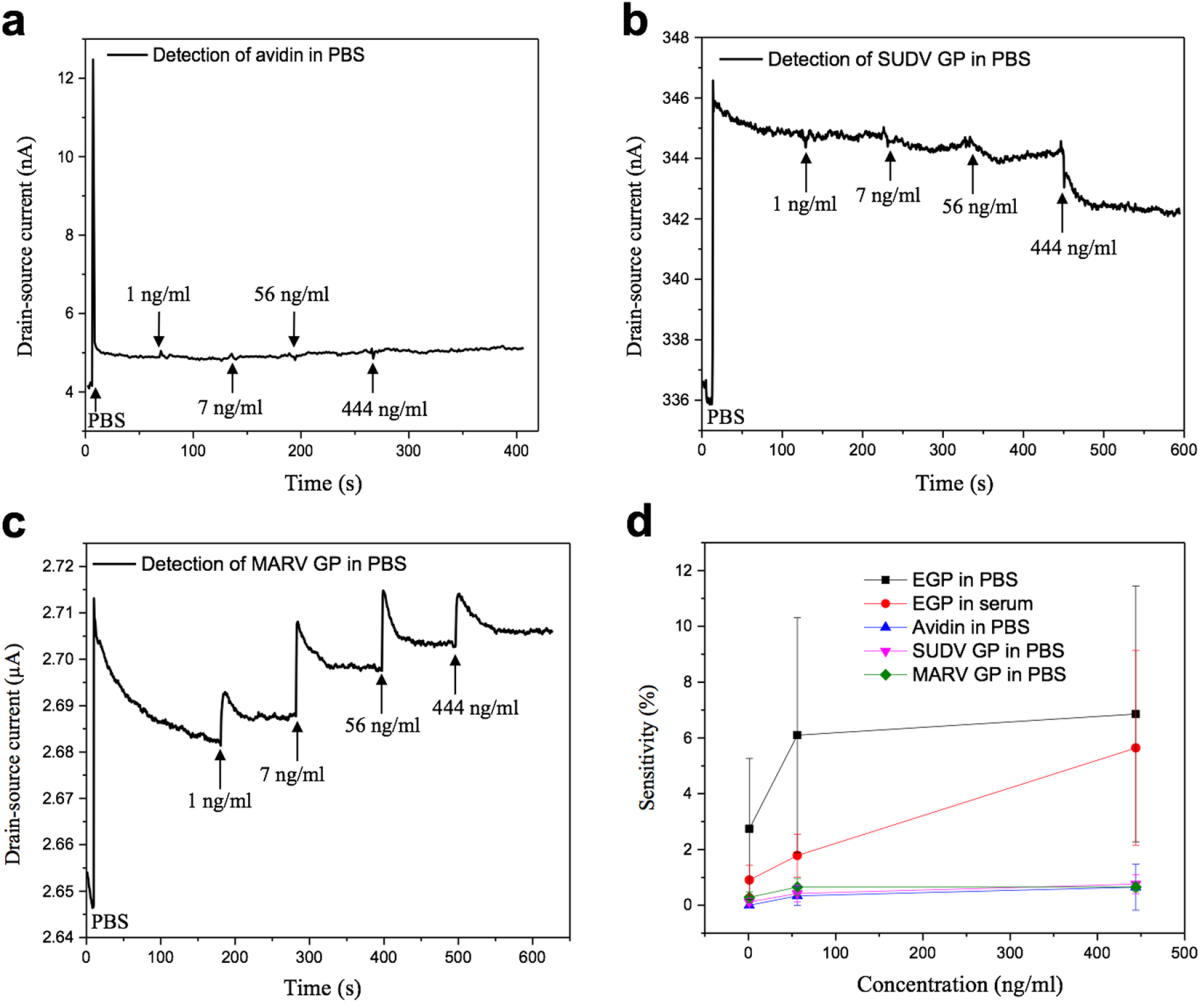
Dynamic response of the FET biosensor towards non-specific proteins suspended in 0.01 × PBS. (**a**) Avidin. (**b**) SUDV GP. (**c**) MARV GP. (**d**) Comparison of sensitivity as a function of protein concentration. The data was collected by five independent replicates. The specific binding of EGP leads to a higher sensitivity compared with other non-specific proteins. For (**a**–**c**), V_ds_ was fixed at 0.01 V.

To demonstrate the high selectivity of the biosensor, control experiments were carried out under the same conditions to study the sensing performance towards avidin, a non-specific protein. After 0.01 × PBS was pipetted onto the device and the baseline was established, a group of avidin samples with increasing concentration was added, and the I_d_ slightly increased, as shown in Fig. 4a, indicating the response from non-specific binding of protein is negligible. To further investigate the cross reactivity, we tested the biosensor’s response towards Sudan virus (SUDV) GP and Marburg virus (MARV) GP. SUDV is within the genus Ebolavirus, which caused several EVD outbreaks in the past; MARV belongs to the Filoviridae family and causes hemorrhagic fever. We measured the dynamic response and sensitivity of the FET biosensors with anti-Ebola probes (specific to the Zaire strain), while samples containing the GP of SUDV and MARV were suspended in 0.01 × PBS. The negative control experiments were carried out under the same conditions as before. The dynamic responses towards these two non-specific GPs are shown in Fig. 4b,c. For 1 ng/ml of SUDV GP and MARV GP, the sensitivity is only about 0.06% and 0.18%, respectively. Therefore, the FET biosensor shows a minimum response to the non-specific GPs and the sensitivity is much smaller than that of EGP, indicating that the cross reactivity is negligible and the sensor has high selectivity to the target protein. A comparison (with error bars) of the sensitivity for EGP, avidin, SUDV GP, and MARV GP as a function of concentration is plotted in Fig. 4d with five independent replicates. We performed these measurements on different devices. Although the devices had varying responses to the antigen due to device-to-device variations, all devices showed a diminished drain current when exposed to EGP. The sensitivity for EGP in 0.01 × PBS is significantly higher than those of non-specific proteins, thereby demonstrating that the probes can specifically capture the target protein and thus change the I_d_. Meanwhile, the sensitivity for EGP in 0.01 × serum also is lower than that in 0.01 × PBS, but still higher than the negative control groups. The performance of the FET biosensor in detecting Ebola antigen is compared with other published works/commercial products in Table 1. Obviously, the FET biosensor has the advantages of a lower detection limit (1 ng/ml of EGP) and shorter processing time (within a few seconds).

**Table 1:** Comparison of the performance on Ebola antigen detection between the current FET biosensor and other published works/commercial products. For limit of detection, some studies utilized pfu/ml as a measure of virus concentration, while other studies including ours utilized ng/ml for protein concentration. There is no direct conversion between the two units.

	Technique	Target	Dispersion medium	Limit of detection	Processing time
Our work	FET	EGP	PBS, serum, plasma	1 ng/ml	within a few seconds
ReEBOV^TM^	chromatography	VP40	blood, plasma	625 ng/ml	15–25 min
Duan *et al*.^9^	chromatography	EGP	serum	100 ng/ml	30 min
Yen *et al*.^8^	chromatography	EGP	serum	150 ng/ml	
Daaboul *et al*.^10^	single-particle interferometric reflectance imaging sensor	pseudotyped Ebola virus	blood, serum	5 × 10^3^ pfu/ml	2 h
Yanik *et al*.^12^	opto-fluidic nanoplasmonic	pseudotyped Ebola virus	PBS	106 pfu/ml	90 min
Cai *et al*.^11^	opto-fluidic chip	Ebola RNA	water	0.2 pfu/ml	3–10 min

## Discussion

For our FET sensor platform, the channel material (i.e., rGO here) bridges the source and drain electrodes. The conductance of the channel is tuned by the external V_g_ applied to the gate oxide (i.e., gating effect). On top of the rGO sheet, the FET sensor consists of an Al_2_O_3_ layer, gold NPs, and antibody probes. The Al_2_O_3_ layer plays two roles here: on one hand, it works as a passivation layer to protect the underlying rGO from degradation when exposed to ambient conditions; on the other hand, it works as the top gate oxide. Gold NPs are used mainly to anchor the antibody probes, which specifically capture EGP. It should be pointed out that the antibody here can also be viewed as a dielectric medium.

To elucidate how the source-drain current changes, we must first understand the net surface charge of EGP. The isoelectric point for EGP is around 6.4, according to the technical data from the vendor, indicating that the molecules carry negative charges in 0.01 × PBS (pH ~ 7). The Zeta potential was measured by dynamic light scattering (DLS, Zetasizer Nano ZS, Malvern) as ~8 mV, which further confirmed that the EGP molecules carry negative surface charges. The antibody probes can specifically pick up negatively charged EGP in the sample and the captured molecules induce a positive potential on the gold NPs through the dielectric antibody. The positive potential (equivalent to positive V_g_) of the gold NPs then leads to the decreased hole concentration in the p-type rGO through Al_2_O_3_ gate oxide, leading to a decrease in the electrical conductance of the rGO.

A major challenge for all diagnostic methods to confirm the presence of Ebola virus is the low levels of viral RNA and antigen in the early stage of an infection. Thereafter, however, the virus increases logarithmically and is particularly elevated in patients dying of Ebola. It is now known that the Ebola virus can persist in human tissues for much longer than the average 21 days’ course of clinical disease, and rarely has been reported to spontaneously reemerge in patients who had cleared the virus. Thus, multiple rounds of blood tests may be necessary to confirm the diagnosis and/or monitor clearance of the virus. Under these circumstances, assay costs and laboratory infrastructure startup costs become even more important considerations. The FET biosensor technology reported here offers advantages of a low assay cost and a low infrastructure startup cost, attractive for Ebola diagnostics in humans and for environmental surveillance^37–39^.

In summary, we developed an rGO-based FET biosensor for rapid detection of the Ebola antigen. rGO sheets were attached on the electrodes as the conducting channel, with anti-Ebola probes immobilized on the surface to capture the specific antigen. The device showed excellent semiconductor characteristics and could be used to detect EGP down to 1 ng/ml with a real-time response. The FET biosensor also has high selectivity towards EGP, with negligible responses from non-specific proteins. To explore the feasibility of using the FET biosensor for diagnosing EVD patients, we tested the sensor’s performance with antigens suspended in 0.01 × PBS/human serum/plasma. The experimental results demonstrated that the FET biosensor could be a candidate for rapidly screening EVD patients in early stages of the disease, with extra benefits of low-cost and ease of use. Further study will focus on improving the sensor’s performance, especially for the plasma samples, as a possible solution for EVD control.

## Methods

### Materials

GO water suspension was ordered from ACS Materials. Cysteamine, PBS, Avidin, and glutaraldehyde were ordered from Sigma-Aldrich. SuperBlock Tween 20 was ordered from Thermo Scientific. Human anti-EBOV glycoprotein monoclonal antibody, EGP, SUDV GP, and MARV GP were ordered from IBT Bioservices.

### Device fabrication

Gold interdigitated electrodes with a gap of 1.5 μm were fabricated using a maskless aligner (MLA-150, Heidelberg Instruments) to pattern the electrodes on a silicon wafer with a top layer of SiO_2_ (thickness ~200 nm), followed by gold deposition on the patterns. Cysteamine solution (1 mg/ml) was pipetted onto the sensing area to functionalize the finger electrodes for 30 min, while cysteamine molecules were assembled/adsorbed on the surface of the electrodes. Surface-modified electrodes were immersed in GO (0.1 mg/ml) water suspension for 30 min as the GO sheets were chemically adsorbed on the electrodes. Then, the electrodes were dried and annealed under Argon atmosphere at 400 ^o^C for 10 min to thermally reduce the attached GO sheets. In order to passivate the surface of the obtained rGO, a thin layer of Al_2_O_3_ (3 nm) was grown on the device using atomic layer deposition (ALD, GEMStar XT, Arradiance). Gold NPs were then deposited on the surface passivated rGO sheets using a sputter coater (SPI Module Sputter Coater) with a gold target for the subsequent antibody immobilization. Cysteamine solution was applied on the sensing area again to functionalize the surface of the gold NPs for 30 min, followed by glutaraldehyde solution (5% in water) for another 30 min. Ebola antibody (0.2 µg in 1 µL PBS), which can be used for detection of EGP and virus-like particles, was then applied on the device to conjugate with the gold NPs and the entire device was incubated at room temperature for 30 min to immobilize the antibody probes. Before testing the sensor performance with EGP, the devices were incubated with blocking buffer (0.1% SuperBlock Tween 20 in PBS) for one hour to avoid the non-specific binding of analytes.

### Device characterization

A Hitachi S-4800 UHR FE-SEM was used to characterize the rGO-based FET device with an acceleration voltage of 2 kV. Atomic force microscopy was conducted by Agilent Technology 5420 AFM with a cantilever (Nanosensors PPP-NCH). A Keithley 4200 semiconductor analyzer was used to characterize the electrical characteristics of the FET devices. The direct current measurement was conducted by measuring the I_d_ with V_ds_ sweeping from −2.0 V to + 2.0 V. In the transistor measurement, I_d_ was recorded by the analyzer with a fixed V_ds_ (0.01 V), while V_g_ sweeping from −40.0 V to + 40.0 V. The dynamic response was studied by monitoring I_d_ with a fixed V_ds_ (0.01 V), while samples containing different concentrations of protein were drop-cast onto the device. All measurements of EGP were carried out under the ambient room temperature condition. The change of I_d_ indicates the presence of EGP compared with the negative controls. Each group was replicated with five independent measurements to study the repeatability.
